# Low-Dose Micro-CT Imaging for Vascular Segmentation and Analysis Using Sparse-View Acquisitions

**DOI:** 10.1371/journal.pone.0068449

**Published:** 2013-07-01

**Authors:** Bert Vandeghinste, Stefaan Vandenberghe, Chris Vanhove, Steven Staelens, Roel Van Holen

**Affiliations:** 1 Institute Biomedical Technology, MEDISIP, Ghent University-iMinds, Ghent, Belgium; 2 Molecular Imaging Center Antwerp, University of Antwerp, Wilrijk, Belgium; University of Navarra, Spain

## Abstract

The aim of this study is to investigate whether reliable and accurate 3D geometrical models of the murine aortic arch can be constructed from sparse-view data in vivo micro-CT acquisitions. This would considerably reduce acquisition time and X-ray dose. In vivo contrast-enhanced micro-CT datasets were reconstructed using a conventional filtered back projection algorithm (FDK), the image space reconstruction algorithm (ISRA) and total variation regularized ISRA (ISRA-TV). The reconstructed images were then semi-automatically segmented. Segmentations of high- and low-dose protocols were compared and evaluated based on voxel classification, 3D model diameters and centerline differences. FDK reconstruction does not lead to accurate segmentation in the case of low-view acquisitions. ISRA manages accurate segmentation with 1024 or more projection views. ISRA-TV needs a minimum of 256 views. These results indicate that accurate vascular models can be obtained from micro-CT scans with 8 times less X-ray dose and acquisition time, as long as regularized iterative reconstruction is used.

## Introduction

Mouse models provide valuable information about the development and progression of cardiovascular pathologies within a reasonable timeframe. Genetically modified mice can be used to study abdominal aortic aneurysm formation [1], cerebral aneurysm formation [2], atherosclerosis [3], vascular remodeling [4], angiogenesis [5], or even specific genetic disorders such as Marfan and Loeys-Dietz syndrome [6], [7].

In order to perform such research, reliable 3D models of the cardiovascular system have to be made. These models are then used as input for Computation Fluid Dynamic (CFD) simulations. *Ex vivo* techniques using vascular corrosion casting [8] proved to be a good tool, resulting in high quality models [9]. However, these *ex vivo* techniques eliminate the possibility to gather longitudinal information, which is considered crucial in evaluating pathology evolution and in therapy development [10].

With the advent of improved CT contrast agents for specific preclinical usage and improved micro-CT systems, it became possible to obtain those high quality 3D models using *in vivo* imaging techniques. In Vandeghinste et al. [11], we compared the accuracy of *in vivo* models and the parameters derived to *ex vivo* reference models. We found that *in vivo* micro-CT allows one to build reliable 3D geometrical models of the cardiovascular system, making follow-up studies possible.

One major difficulty in longitudinal aneurysm studies is the total number of scans needed to characterize the aneurysm formation process fully. Because the onset of the aneurysm development is not known and varies greatly per individual animal, daily CT scans need to be taken in hopes of having data just before and just after the start of the formation. Secondly, there is a possibility of aneurysm dissection or rupture in a later stage, with the most important information available just before the dissection. This information cannot be obtained anymore after the dissection. Daily scans over the course of weeks during such longitudinal studies will result in a large X-ray dose.

Unfortunately, little is known about the potential side effects of the dose on preclinical studies. The effects of animal strain, age, radiation location and the many measurement protocols available result in a large number of combinations to study [12]. Nevertheless, Laforest et al. [13] have shown that total doses of 0.180 Gy can already lead to tumor growth inhibition in mice. Klinck et al. [12] showed how weekly exposure to high-resolution micro-CT reduces the trabecular bone volume 8 to 20% in skeletally immature BALB/cByJ and C57BL/6J mice. Others have presented results to the contrary [14]. Nevertheless, using the lowest possible radiation would help remove any doubt about negatively influenced results [15], as typical X-ray whole body radiation doses from 3D micro-CT scanners range from 0.017 to 0.78 Gy [16], well in the range of the studies described above.

A second issue is the throughput. With daily scans, the throughput needs to be maximized to efficiently plan these studies amongst others. This necessitates fast acquisition protocols and fast reconstruction algorithms.

One possibility to achieve higher throughput and lower dose at the same time is reducing the number of projection views acquired during the scan. This allows for a faster rotation time and thus reduces the dose-time-product proportionally. If a filtered back-projection (FBP) type algorithm is used to reconstruct these datasets, the images will show streaking artefacts [17]. This is due to the limited amount of data being available. Even though iterative reconstruction algorithms allow for much more accurate modelling of the acquisition system and physics, streaking artefacts will not be completely eliminated.

One of the techniques that have been extensively investigated to solve the problem of limited data is iterative CT reconstruction combined with Total Variation (TV) minimization. TV minimization has been used before for few-view, limited angle reconstruction [17]–[25], algorithm-enabled low dose imaging [26], and CT image denoising or restoration [27], [28]. It assumes that medical images can be conceptualized by edges and uniform intensities [29], [30]. After applying the gradient operator, the image is transformed into an edge map, which only contains a small number of significant coefficients. TV regularization will then search for the reconstructed image that is optimally sparse, i.e. has the least number of significant coefficients. This may ultimately result in a cartoon-like image, with minimal noise. Although cartoon-like images are not desired for diagnostic CT imaging [18], they may still offer enough information for segmentation purposes and dose reduction.

The aim of this study is to investigate whether reliable and accurate 3D geometrical models of the murine aortic arch can be reconstructed using few view *in vivo* micro-CT acquisitions. By reducing the number of projection views, both the acquisition time and the dose will decrease proportionally. With conventional reconstruction methods this would introduce excessive noise. We reduce the noise and artefacts using TV minimization. We compared geometrical models obtained using sparse view acquisitions with models obtained from full-view acquisitions of the same animals.

## Materials and Methods

### Ethics statement

Data from a previous study [11] were used, as per the guidelines of ethical practice in animal experimentation. No additional animal work was conducted.

### Data acquisition

Four wild-type mice were administered an iodine-based contrast agent (Fenestra VC-131, Advanced Research Technologies Inc., Saint Laurent, Canada) with a dose of 15 µl/g to enhance the vasculature on the images. The animals were scanned 25 minutes post injection, to achieve optimal image contrast. The acquisitions were measured on a FLEX Triumph CT scanner (TriFoil Imaging, Northridge, USA) with the following acquisition parameters: 50 µm focal spot, 2×2 detector binning, 2048 projections over 360 degrees, 3.5 times magnification and 70 kVp tube voltage. The geometrical parameters result in a 33.81 mm field of view, a theoretical spatial resolution of 46 µm and a scanning time of 8.53 minutes. The ideal tube current, which utilizes the dynamic range of the detector optimally, was determined at 180 µA for a 70 kVp tube voltage. According to another study on the same system [15], these settings would result in a dose of about 0.12 Gy per scan.

### Reconstruction methods

The measured data were retrospectively subsampled to simulate dose reduction. Seven distinct datasets were generated from the measured projection data, by removing all but every *n*th projection to obtain datasets with 2048/*n* uniformly spaced projection views. In this way, datasets were obtained with 1024, 512, 256, 128, 64 and 32 views over 360 degrees. Based on literature [15], these views would thus correspond to nominal dose levels of 60, 30, 15, 7.5, 3.75 and 1.875 mGy. The dataset with all 2048 projection views (nominal dose 120 mGy [15]) can then serve as a reference dataset to these few-view datasets.

The datasets were reconstructed using 3 different algorithms: image space reconstruction algorithm (ISRA), ISRA with TV regularization (TV) and FBP. ISRA is an adaptation of the maximum likelihood (ML) algorithm, and results in non-negative reconstructed images [31], [32]. Mathematically, the algorithm is described by



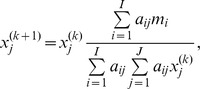
with *I* and *J* respectively the number of detector pixels and voxels, 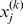
voxel j at iteration *k*, 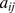
the contribution of voxel *j* to detector *i*, and vector 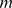
the measured data.

**Figure 1:**
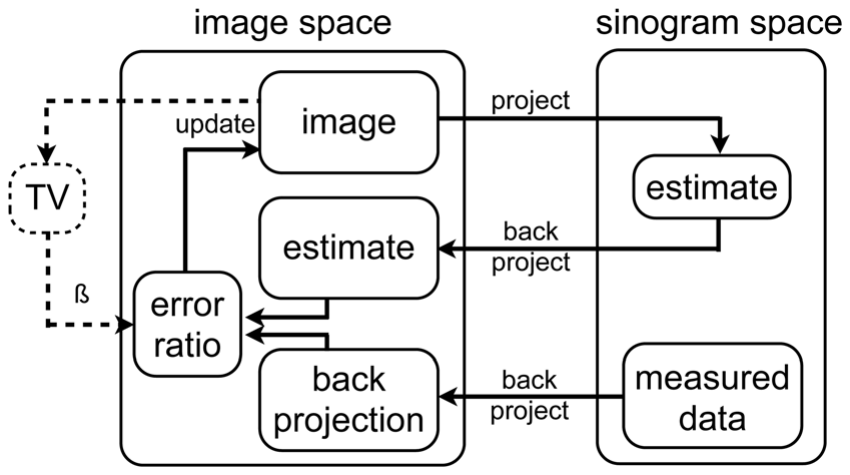
ISRA and ISRA-TV in block diagram. Block diagram to show how data flows through ISRA (full lines) and ISRA-TV (full + dotted).

Its implementation is easy and straightforward, and is depicted in Fig. 1: first the measured data *m* is back-projected and stored. Next, the current image estimate *x_j_* is forward and then back-projected, and the ratio of the back-projected data *m* and this image estimate is calculated. Finally, this ratio is used to multiplicatively update the current image. This type of algorithm is ideal to reconstruct large preclinical datasets, as the sinogram *m* is only used to calculate the back-projection of the measured data once. From then on, only forward and back-projections are needed, without comparing to the measured data again in sinogram space. This results in a considerable decrease in computation time, because the large sinogram is read from the hard drives only once.

The second algorithm is implemented by adapting ISRA with a one-step-late (OSL) [33], [34], [35] modification to incorporate the TV-norm. The modification is based on Lange [34] and Defrise et al. [35], where TV was incorporated in emission and transmission tomography algorithms. When only a small number of projection views are used, it becomes much more difficult to solve the system of equations, as this system becomes more and more underdetermined. TV regularization will act as a penalty, enforcing a sparse image gradient. In other words, the image will be forced towards being uniform between edges. The dashed lines in Fig. 1 represent the TV part that is added to the ISRA method. Here, the TV calculated on the previous image iteration is used to change the error ratio for the next image iteration. The degree of regularization can be varied with one relaxation parameter 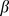. The ISRA-TV algorithm can be described by



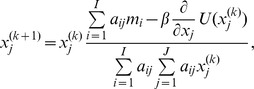
with 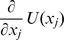 the partial derivative of the energy function *U* [34], [36]:
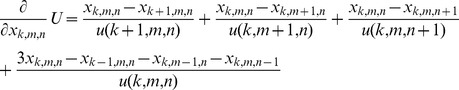





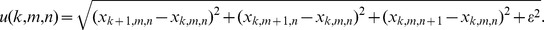



Parameter 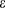 is a small positive number to resolve the discontinuity at zero in the derivative of the TV.

The implementation of the iterative algorithms was done on the GPU in CUDA. The forward projector was implemented using a pixel-driven approach, by connecting a detector pixel with the location of the X-ray tube and calculating image samples along this ray. The samples can be easily fetched using the 3D texture interpolation facilities available in CUDA. The back projector uses a voxel-driven approach, which connects each voxel with the X-ray tube and calculates the value at the intersection of this ray with the detector. This can be done using 2D texture interpolation. The finite size of the focal spot was not modelled. Furthermore, no ordered subset versions of the algorithms were used, as convergence would not be guaranteed in that case [37], [38].

To compare the results obtained with ISRA and ISRA-TV with traditional results, we also reconstructed the data with an FBP-type algorithm for cone-beam systems such as ours: the algorithm of Feldkamp, Davis and Kress (FDK) [39] (Cobra EXXIM, EXXIM Computing corp., Livermore, USA). FDK is a standard reconstruction method and is primarily used for its fast reconstruction result even in large datasets. The algorithm does not employ any advanced modelling to reduce noise encountered by sparse sampling.

All images were reconstructed to a 100 µm voxel matrix. The matrix dimensions were determined per animal to always encompass the whole body. All iterative algorithms were stopped at convergence, defined as 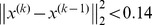. The value of 0.14 was empirically determined. For ISRA-TV, the regularization parameter 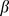 was always set to 0.001. This was selected empirically to provide a good regularization, without overly smoothing.

### 3D Segmentation

In our previous study, the segmentation was done manually in the Mimics software package (Materialise, Leuven, Belgium). The aortic arch was manually thresholded in a first segmentation step. When the resulting mask was judged sufficiently accurate, a 3D geometric model was built according to this mask. This 3D model was then smoothed in Mimics Remesher (Materialise, Leuven, Belgium) to remove anatomically impossible bulges and dents without shrinking the model. The result of these operations was a 3D model sufficiently smooth and simple to be useful in CFD simulations.

Since this manual thresholding approach could lead to subjective results we propose to use a semi-automatic method in this work. To get an objective quantitative measure, the first steps that would be executed during manual segmentation were implemented in a semi-automatic fashion. The semi-automatic segmentation results can then be compared. Although this method does not result in a perfectly segmented aortic arch with inclusion of the carotid arteries and the abdominal aorta, the segmentation result is still representative of a starting point for further manual editing.

As a first step, the semi-automatic segmentation starts with the definition of a volume of interest (VOI) inside the aortic arch. The mean *v*, standard deviation σ, minimum *v_min_* and maximum voxel values *v_max_* are then determined inside this VOI. Next, thresholding is applied to preliminary segment the image with voxel values in the interval [MAX(*v_min_*, *v* – 3σ), MIN(*v_max_*, *v* + 3σ)].

To select the arterial tree we need to apply three steps. First, because noisy voxels may wrongly associate with the arterial tree in later steps, the segmentation is eroded by 1 pixel (voxel is replaced with the minimum value of the 26 neighbours in 3D). Next, automatic region growing is started using the aortic arch VOI as seed. Finally, the resulting mask is dilated by 1 pixel (voxel value is replaced with the maximum value of the 26 neighbours in 3D), to regain the volume removed by the erosion operation.

**Figure 2:**
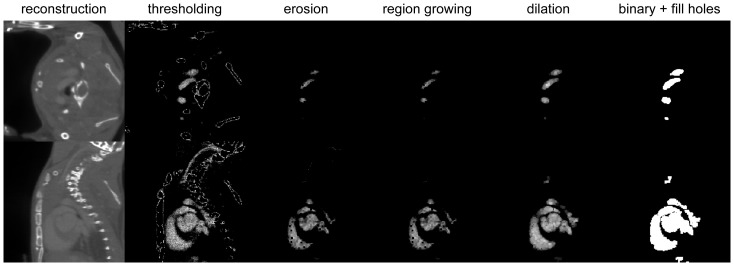
Sub steps of semi-automatic segmentation. Transversal and sagittal slices of each automatic segmentation operation, going from the reconstructed dataset on the left to the final binary image on the right.

As a last operation, the segmentation is converted to a binary image and the remaining holes are filled (binary OR operation of the voxel value with its 26 neighbours). The result of each operation is depicted in Fig. 2 for a full-view (high-dose) dataset.

### Analysis

To compare the reconstructions of each few-view dataset, the full-view reconstruction was first automatically segmented to obtain a reference segmentation mask, serving as the gold standard segmentation. For ISRA and ISRA-TV, the ISRA reconstruction from the 2048-view dataset is used as a reference. For FDK, the FDK reconstruction from 2048-view dataset is used. The sparse-view segmentations were evaluated based on 3 values: the amount of correctly classified voxels or true positives (TP), the amount of not segmented voxels or false negatives (FN) (segmented on the full-view but not on the few-view reconstruction), and the amount of additionally segmented voxels or false positives (FP) (segmented on the few-view but not on the full-view reconstruction).

**Figure 3:**
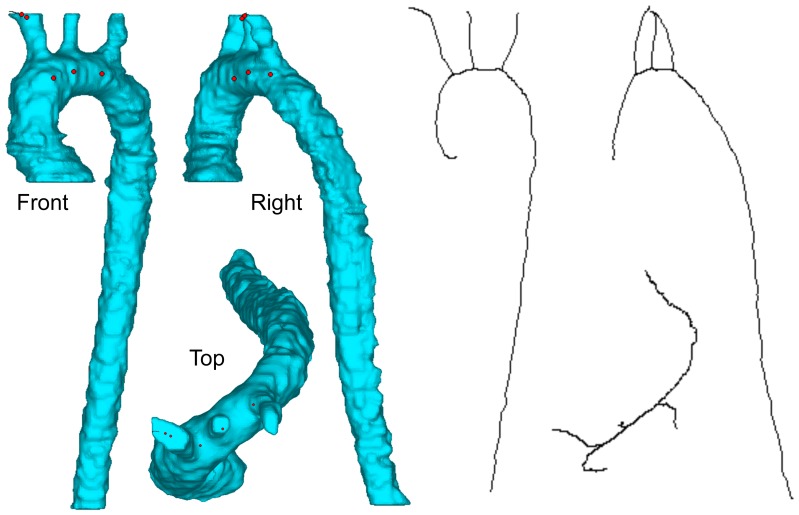
Segmentation mask and centerline. Front, right and top view of the aorta segmentation mask and the resulting centerline.

To compare the segmentations in more detail, the aorta diameters and aorta positions were compared. First a centerline was fitted to the aorta mask using the MedCAD module of Mimics. Because this centerline cannot be calculated when the segmentation still includes parts of the heart, all masks had to be manually edited to only retain the aorta. This was done blinded for all datasets. The 2048-view ISRA dataset was manually edited twice: once to serve as a reference centerline and once as part of the blinded datasets. The difference between both centerlines then allows us to investigate the error due to the manual adjustments. Figure 3 illustrates the shape of the resulting aorta segmentation next to its calculated centerline. No data of FDK reconstructions was included in this part of the study, as that data was reconstructed with proprietary software. Careful comparison between the FDK reconstruction and our iterative software showed sub-voxel differences. This led to a considerable centerline offset, which does not represent changes due to sparse-viewing artifacts, but due to mismatched registration.

For each 3D centerline control point, the closest point (in L2-norm sense) on the reference centerline was searched. The error on the aortic diameter was then calculated by averaging the relative error between the diameter *d_ref_* of the reference segmentation and the diameter *d* of the segmentation in question over each pair of control points *p*: 
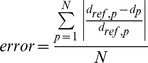



Each individual diameter was calculated as the best-fit diameter, fit on the plane orthogonal to the centerline going through the control point. The offset between both centerlines was calculated by calculating the average 3D Euclidean distance between this centerline and the reference centerline for all pairs of control points.

Student's T-test was used to test the results of different datasets for significance to the error seen with 2048-view ISRA. As the 2048-view ISRA results are segmented twice, this allows us to measure the error due to manual editing to retain the aorta. A significant (p<0.05) difference with this error then signifies errors not caused by manual editing.

## Results

**Figure 4:**
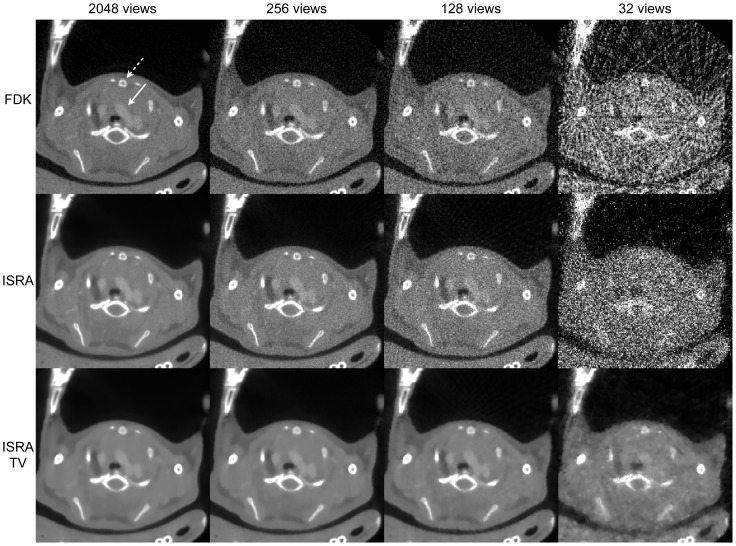
Transversal reconstruction results. Transversal view of reconstructions for FDK (top row), ISRA (middle row) and ISRA-TV (bottom row) for different amounts of projection views. Full arrow points to the aortic arch. Dashed arrow points to the sternum.

**Figure 5:**
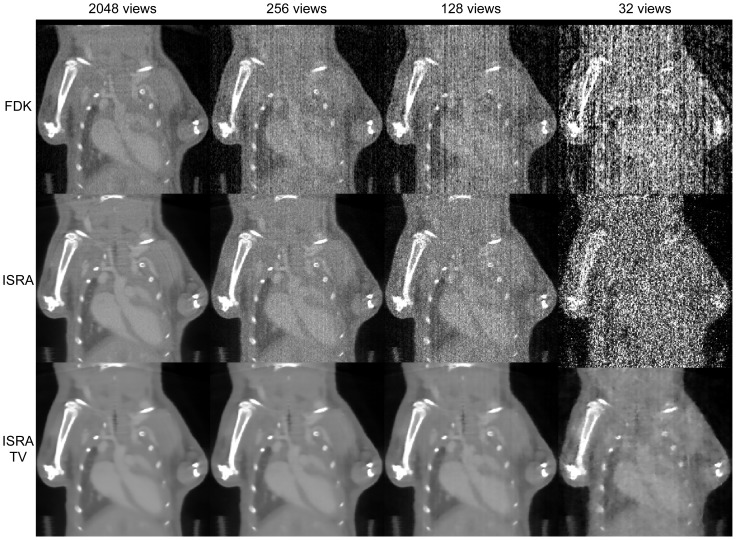
Coronal reconstruction results. Coronal view of reconstructions for FDK (top row), ISRA (middle row) and ISRA-TV (bottom row) for different amounts of projection views.

Fig. 4 and Fig. 5 depict the images obtained at different few-view levels, corresponding to nominal whole-body dose levels as extrapolated from literature [15] of 120 mGy, 15 mGy, 7.5 mGy and 1.875 mGy. Conventional FDK and ISRA lead to excessive image noise for lower number of views, reducing the visibility of the aortic arch (full arrow in Fig. 3). While ISRA-TV reduces the streak artefacts considerably when only 256 and 128 views are used, it does not manage to sufficiently reduce the artefacts when only 32 views are used. Furthermore, a small loss of spatial resolution can be noted in all ISRA-TV reconstructions, compared to ISRA without regularization. This is apparent by comparing the sternum in the top right on the full-view datasets (dashed arrow in Fig. 4). On the other hand, the coronal view in Fig. 5 shows that some vessels in the lung can still be identified at 128-view ISRA-TV, while they are obscured by noise for ISRA and FDK.

**Figure 6:**
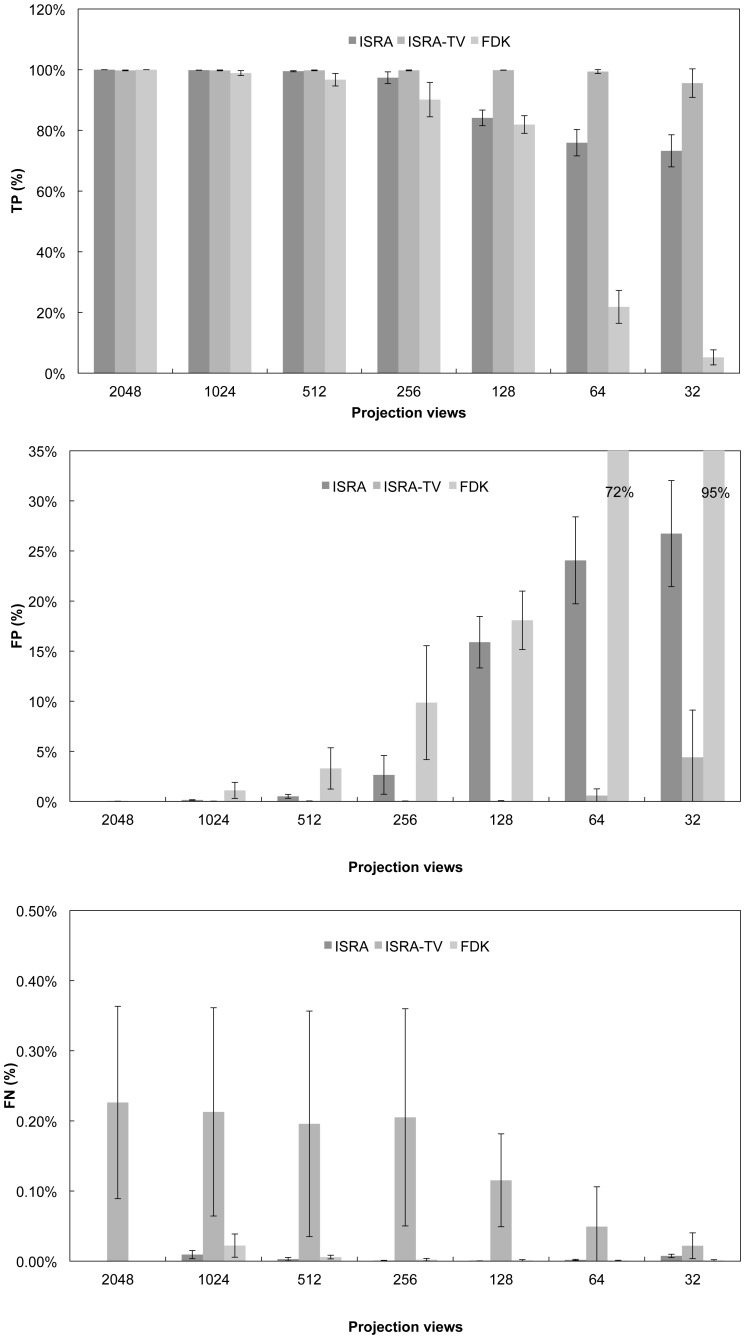
Quantitative comparison between different algorithms. TP, FP and FN for each algorithm. Note that each plot has a different scale, which is very low in the case of FN.

Fig. 6 shows the classification accuracy when the amount of views is reduced. The error bars represent the standard deviation over the animals. ISRA-TV manages correct classification with 2048 and 1024 view datasets, which also holds for ISRA when 1024 views are used. Starting from 512 views on, a trend of overestimation (higher FP fraction) occurs for ISRA and FDK. This trend is not seen with ISRA-TV, which remains correct as long as more than 64 views are used. A small underestimation is always present with ISRA-TV, not seen with ISRA nor FDK. The FDK reconstruction already shows some overestimation at 1024 projection views, reducing the number of correctly classified voxels.

**Figure 7:**
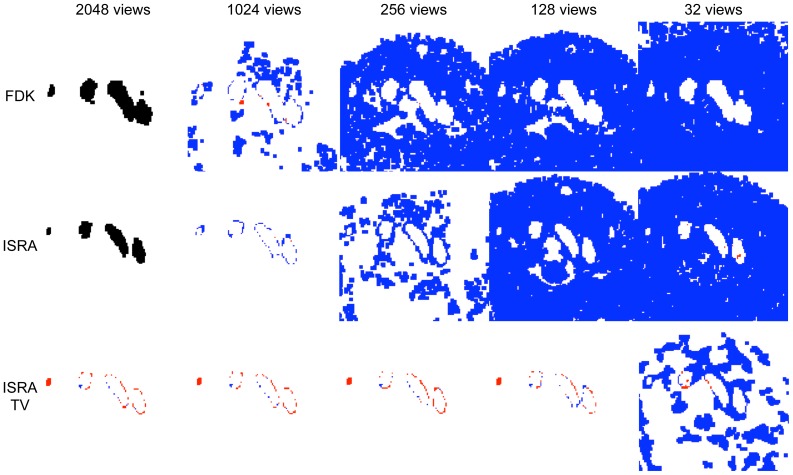
Difference images through the aortic arch. Colour coded difference images of one transversal slice through the aortic arch. Black is reference, white is TP, blue is FP, red is FN.

**Figure 8:**
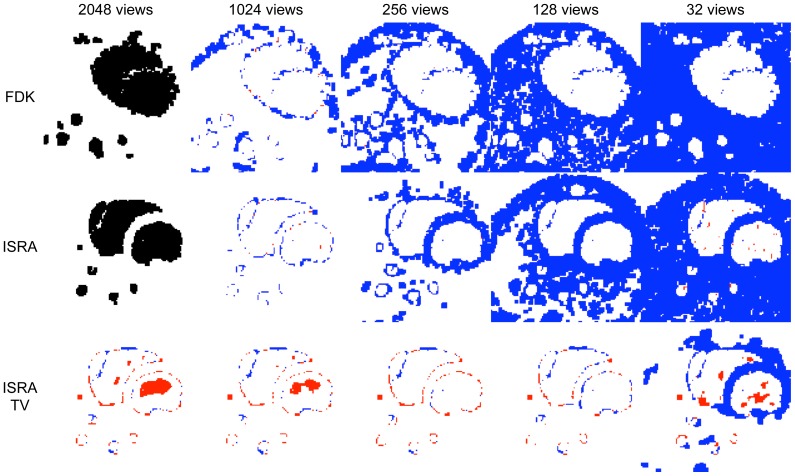
Difference images through the heart. Colour coded difference images of a transversal slice through the heart. Black is reference, white is TP, blue is FP, red is FN.

Fig. 7 and 8 compare the reconstructions and segmentations of 2048, 1024, 256, 128 and 32 views FDK, ISRA and ISRA-TV on one transversal slice. The segmented image is colour coded, showing white where there is correct classification (be it as background, or as part of the arterial tree), blue when there is overestimation and red when there is underestimation. The black segmentation mask is the reference mask obtained from the high dose FDK reconstruction for all fewer-view FDK reconstructions, or from the high dose ISRA reconstruction for all fewer-view ISRA and ISRA-TV reconstructions.

The FDK reconstructions do not lead to an accurate segmentation, even when 1024 views are available. The large shape disagreement between the 2048-view FDK and 2048-view ISRA reconstruction is due to the much higher noise in the FDK reconstruction, even though the maximum number of projection views was used.

Both iterative reconstruction methods manage to obtain an accurate segmentation on the 2048 and 1024 view datasets. There is some difference, as ISRA tends to overestimate the segmentation volume (blue), while ISRA-TV both over- and underestimates at the same time. When 256 views are used, ISRA tends to overestimate considerably, increasing the segmentation volume. ISRA-TV is more accurate at 256 and 128 views, and overestimates more when the number of views is further reduced (more blue). Finally, both methods fail segmentation at only 32 views.

Table 1 contains the relative diameter errors compared to the reference centerline, which was calculated separately from 2048-view ISRA. The error for 2048-view ISRA is the error caused by manually adjusting the segmentation. Although the semi-automatically generated masks correctly delineated the abdominal aorta, the delineation between the heart and descending aorta was not always that clear. This means we could sometimes not manually separate the heart and the descending aorta without doubt about changes to the aorta mask. These datasets were not included in the comparison. Using 512 views with ISRA leads to a significantly larger diameter error (p<0.05) than for 2048-view ISRA. The mean aorta diameter seen on the reference segmentations is 1.2 mm. The 13.7% error for 512-view ISRA is thus equal to a difference of 164 µm. When TV is used to reduce the noise and image artifacts, the diameter errors do not change significantly, even when only 256 views are used.

**Table 1:** Relative aorta diameter errors.

views	ISRA	ISRA-TV
**2048**	5.10±1.24%	7.22±1.19%
**1024**	6.56±2.32%	7.97±1.09%
**512**	13.66±3.37% *	7.59±1.94%
**256**	-	6.52±1.60%
**128**	-	-
**64**	-	-
**32**	-	-

Comparison of relative aorta diameter errors (mean error ± SD, n = 4) *: Significance by comparing to 2048-view ISRA (p<0.05). Manually nonadjustable data denoted with -.

Table 2 details the centerline distance between the different methods. Again, the distance for 2048-ISRA is the distance caused by manually-adjusting the segmentation to enable the centerline calculations. When fewer views are used with ISRA, the centerline offset does not change significantly. However, this does not hold for 512-view and 256-view ISRA-TV, where a significant (p<0.05) different distance can be found compared to the distance expected from adjusting the segmentation manually. The highest error can always be found at the top of the aortic arch, where the carotid arteries influence the centerline calculation.

**Table 2:** Centerline distance.

	ISRA	ISRA-TV
views	distance (mm)	distance (mm)
**2048**	0.046±0.011	0.064±0.014
**1024**	0.054±0.009	0.075±0.022
**512**	0.059±0.002	0.070±0.014 *
**256**	-	0.065±0.008 *
**128**	-	-
**64**	-	-
**32**	-	-

3D Euclidean distance between centerlines (mean ± SD, n = 4). *: Significance by comparing to 2048-view ISRA (p<0.05). Manually nonadjustable data denoted with -.

**Figure 9:**
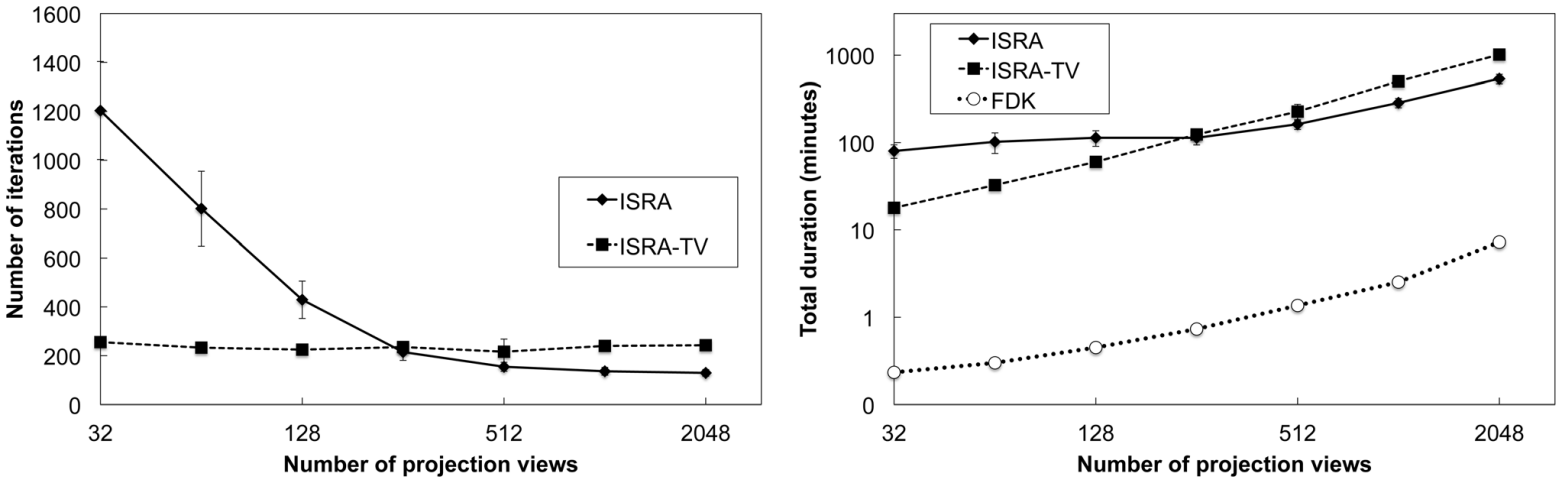
Reconstruction time needed to reach stopping criterion. (left) Number of iterations needed to reach the stopping criterion. (right) Total reconstruction time needed to reach the stopping criterion (logarithmic scale).

Fig. 9 plots the number of iterations needed to reach the stopping criterion and the total reconstruction time (logarithmic scale) for both iterative algorithms and per number of used views. As a reference, the total time needed for FDK is also included. Our implementation ran on one Intel Xeon E5620 core (2.4 Ghz) with 32 GB RAM memory, interfacing with one Nvidia Tesla M2070 GPU. The usage of TV regularization leads to a slow-down, doubling the total reconstruction time needed at 2048 views for ISRA-TV compared to ISRA. This is due to the overhead of calculating the TV per iteration. When less than 256 views are used, this slow-down will be out-weighted by the guaranteed and faster halting of the algorithm, which means much less iterations are needed to reach our stopping criterion. For ISRA-TV, the number of iterations needed is independent of the number of projection views and remains constant around 235±13 iterations. At 128 projection views or less, ISRA-TV thus leads to a faster solution compared to ISRA. At 128 views, the reconstruction time for ISRA-TV is already half the time needed by ISRA.

## Discussion

Segmentation accuracy was used as a metric to determine if few-view CT can be used to segment the aortic arch and connected vessels. Based on the results presented, we found that 256-view acquisitions can result in comparable segmentations as full-view acquisitions, as long as TV regularization is used. This leads to an 8-fold reduction in X-ray dose, and an 8-fold shorter acquisition. This leads to a considerable increase in throughput when multiple studies need to be performed on the same day. Using fewer views without using regularized image reconstruction will lead to errors in the vessel diameters. Regularized reconstruction on the other hand will keep the diameters. Although a significant geometry displacement may occur, it is generally smaller than 0.075 mm, which is smaller than the reconstructed voxel size.

FDK is currently used the most in preclinical practice due to its fast reconstruction time and the number of commercially available software packages providing this reconstruction algorithm. However, this comes at a cost, as the segmentation quality is already negatively influenced even at 1024 views. In contrast, iterative reconstruction is much more robust to dose reduction. Non-regularized ISRA also shows its benefits: it can already lead to a 2-fold decrease in dose compared to FDK, or even a 4-fold decrease if a small change in diameter is acceptable.

Although the accuracy of ISRA-TV is very high at high-view settings, both under- and overestimation can be noticed around the aortic arch (Fig. 7). This is consistent with the insignificant diameter loss and the sub-voxel displacement of the centerline. We hypothesize that this is due to resolution loss at the edges. However, this effect was not a limiting factor for our task.

In this study, only a reduction of projection views was employed to reduce the total image dose, as this is the only method retrospectively available. However, other dose reduction methods exist in the clinic [40]. Of these clinical methods, reducing the tube current is the only viable option in preclinical systems. The worse photon statistics will result in an increase in image noise, without inducing the streaking artefacts as encountered in sparse-viewing. We expect comparable results when TV regularization is used on those data sets.

TV reconstruction can be expected to be useful in many of today's pre-clinical studies for higher throughput and dose reduction. Examples are bone imaging to assess bone density during fracture healing [41], bone resorption, remodelling and regeneration [42], bone neoplasms [43], and bone influenced by metabolic disorders such as osteoporosis [44]. Because of the piecewise constant nature of the bone and surrounding tissue, these applications are very suited for TV based reconstruction. Additionally, the nature of these studies primarily requires high-resolution micro CT imaging, making them a good candidate for dose reduction. Other applications may include preclinical PET/CT and SPECT/CT studies, which utilize the CT information for attenuation correction [45], partial volume (PVE) correction [46], and as an anatomical landmark (VOI selection). Currently the same CT image is used as input for these 3 methods. However, especially attenuation correction is a big candidate for ultra-low-dose micro CT scans. Applying TV regularization on our data with only 32 views leads to an inaccurate segmentation of the vessels, but does manage to obtain some visual quality (Fig. 4 and 5). Such images might still be useful for attenuation correction. These low-dose acquisitions can then be co-registered with one higher dose acquisition to generate all information in longitudinal studies.

## Conclusions

This study determined the minimum number of projection views needed to accurately segment the aortic arch and its connected vessels. The same segmentation quality can be obtained as with a high number of views when ISRA-TV is used with a minimum of 256 projection views. This leads to an 8 times decrease in X-ray dose and acquisition time.
